# PAIN-RELATED BRAIN STRUCTURE AND PAIN CATASTROPHIZING IN BLACKS AND WHITES WITH KNEE OSTEOARTHRITIS

**DOI:** 10.1093/geroni/igz038.276

**Published:** 2019-11-08

**Authors:** Ellen Terry, Ellen L Terry, Josue S Cardoso, Kimberly T Sibille, Jared J Tanner, Burel R Goodin, Catherine C Price, Roger B Fillingim

**Affiliations:** 1 University of Florida, Gainesville, Florida, United States; 2 University of Alabama at Birmingham (UAB), Birmingham, Alabama, United States

## Abstract

Pain catastrophizing is a cognitive-affective response during painful experiences and is implicated in the facilitation of pain processing. Non-Hispanic blacks (NHB) more often engage in catastrophizing as a coping strategy compared to non-Hispanic whites (NHW). Hence, pain catastrophizing may contribute to poorer pain-related outcomes and greater disability in NHBs. Functional neuroimaging studies have linked high levels of catastrophizing with increased cerebral responses to pain in the insula and primary somatosensory cortex [S1], but associations between brain structure and catastrophizing remain largely unexplored. Moreover, no neuroimaging studies have investigated whether catastrophizing is differentially associated with pain-related brain structure across racial/ethnic groups. We examined the association between race/ethnicity, catastrophizing, and pain-related brain structure (insula, S1) among 176 participants with and without knee pain. Findings provide evidence for differing associations of catastrophizing with pain-related brain structures in NHBs and NHWs. It is therefore important to develop culturally-relevant, neural-mediated interventions targeting catastrophizing for NHBs.

